# Examining the Use of Mobile Technology to Deliver Tailored Sexual Assault Prevention in a Classroom Environment in the Military: Development and Usability Study

**DOI:** 10.2196/41455

**Published:** 2022-11-16

**Authors:** Randall Eckhoff, Matthew Boyce, Rebecca Lee Watkins, Marni Kan, Nichole Scaglione, Leah Pound, Meghan Root

**Affiliations:** 1 RTI International Research Triangle Park, NC United States; 2 University of Florida Gainesville, FL United States; 3 Headquarters Air Force / A1Z Washington, DC, DC United States

**Keywords:** research techniques, mobile technology, tablet, iPad, restricted, resource limited, Wi-Fi, tailored learning, military, data security

## Abstract

**Background:**

Advances in mobile technology over the last 10 years have expanded its use in scientific research. However, there are challenges in creating a reliable system for intervention content delivery and data collection in an environment with limited internet connectivity and limited staffing capacity. The Sexual Communication and Consent (SCC) study used tablets to provide individualized Sexual Assault Prevention and Response training in a classroom environment that was both technologically and support staff limited.

**Objective:**

We developed the SCC Basic Military Training app and a separate Sexual Assault Response Coordinator app to support individualized training within the new SCC program. This paper presents the functionality, protocols, challenges, and feasibility of deploying mobile technology in an educational environment in the military with limited resources.

**Methods:**

We developed both mobile apps targeting the Apple iOS ecosystem. The Basic Military Training app provided a screening instrument that routed the trainee into 1 of 5 specific intervention programs. Over 2 days of basic military training set 2 weeks apart, trainees received a combined 6 hours of program-specific tablet training, combined with universal, interactive classroom training, led by qualified instructors. The Sexual Assault Response Coordinator app, used to deliver supplemental content to a subgroup of trainees, was made available for voluntary and private use at the Sexual Assault Response Coordinator’s office on base. All anonymous data were manually transferred onto laptops, where the data were aggregated into files and securely transferred to the project staff for analysis. The study was conducted at the Lackland Air Force Base, Joint Base San Antonio, with 9196 trainees providing the data.

**Results:**

A total of 7742 trainees completed both the sessions of the SCC program and a series of evaluative assessments. Some trainees did not receive day 2 training, and only received day 1 training because the COVID-19 pandemic shortened the study period. Of the 190 SCC classes taught, only one class was unable to complete tablet training because of Apple licensing–related technology failure. The 360 study tablets were distributed across 3 classrooms (120 per classroom) and were handled at least 16,938 times with no reports of breakage or requiring replacement. Wi-Fi access limitations exacerbated the complexity of Apple licensing revalidation and the secure transfer of data from the classroom to project personnel. The instructor staff’s limited technical knowledge to perform certain technical tasks was challenging.

**Conclusions:**

The results demonstrated the feasibility of deploying a mobile app for tablet-based training in a military educational environment. Although successful, the study was not without technical challenges. This paper gives examples of technical lessons learned and recommendations for conducting the study differently, with the aim that the knowledge gained may be helpful to other researchers encountering similar requirements.

## Introduction

### Background

Sexual assault continues to significantly impact military force readiness and lethality [1]. The 2018 Workplace and Gender Relations Survey reports an increase in sexual assault from 2016, with 20,473 active-duty service members experiencing sexual assault between October 1, 2017, and September 30, 2018 [2]. In addition to an estimated 16,000 manpower years lost because of sexual assault and sexual harassment each year [3], victims of sexual violence may experience repeat victimization, posttraumatic stress disorder, depression, and anxiety [4,5].

In the military, other service members perpetrate most sexual assaults; both men and women are sexual assault victims, and revictimization is more likely if individuals are sexually victimized before joining the military [2,6-8]. Scientific studies have shown better outcomes from personalized and tailored instruction over one-size-fits-all content [9], including enhanced sexual assault intervention effectiveness [10]. However, tailored programs that address military-specific risk factors do not exist.

The Sexual Communication and Consent (SCC) project aimed to develop a tailored approach to sexual assault prevention with the goal of improving the existing one-size-fits-all approach at the US Air Force Basic Military Training (BMT). The SCC project used modern mobile technology and digital content to provide a tailored learning experience to trainees [11]. With the prevalence of mobile technology in use today, it is only natural to see increased adoption in learning institutions, especially as new learning innovations and technologies emerge [12].

Similar to higher education institutions that are embracing these new learning innovations and technologies, federal institutions such as the military must also begin to do so. The world at present is accustomed to constant digital connectivity, with information accessible anytime and anywhere. By design, mobile technology is expected to be connected to facilitate information sharing even if the information is sensitive and protected. The military has embraced the use of mobile technology in some areas such as resilience strategies [13]; however, these mobile apps run on personal mobile devices. The use of mobile technology within an organization is governed by the rules of that organization. In addition, technologies that consumers take for granted are restricted in some organizations, such as the military, in the interest of national security.

This paper focuses on the lessons learned using modern mobile technology in a restricted learning environment and does not discuss the science behind the SCC study. Broader descriptions of intervention content development, SCC implementation feasibility, and preliminary program outcomes are forthcoming.

### An Overview of the SCC Project

The SCC training included a mixed classroom experience combining instructor-led sexual assault educational activities and discussion with a tailored learning experience using mobile technology to be used during BMT conducted at the Lackland Air Force Base, Joint Base San Antonio, Texas. Early in the development of this training approach, we learned that new trainees were not allowed to access personal mobile devices during basic training, and the training rooms had limited internet access. To facilitate the delivery of tailored training components, we developed 2 offline mobile apps, that is, the BMT app and the Sexual Assault Response Coordinator (SARC) app, which trainees would access on project-provided classroom sets of tablets [11].

The BMT app is the primary app used in the BMT classroom setting. With over 100 trainees per class, and the training split across 2 days, each trainee used the BMT app to first determine their sexual assault risk profile, which was identified from their responses to an anonymous screening assessment. On the basis of their sexual assault risk profile, the trainee then received tailored content from 1 of 5 programs: Revictimization Prevention (separate programs for male and female), Primary Prevention and Situational Awareness Enhancement (separate programs for male and female), and Healthy Relationships and Airmen Intervention (for males with no victimization history or risk). The tailored training was delivered confidentially using the BMT app, and tablet privacy screens and headphones were provided to ensure privacy. The tailored content included videos depicting scenarios in which sexual assault can occur and modeled behavioral strategies that can be used to decrease the risks associated with such situations.

The tailored tablet training was combined with interactive classroom training instruction and activities, led by 2 trained instructors in a 120-minute block on day 1 and a 240-minute block on day 2, for a total of 6 hours of training for each trainee. On day 1, a total of 93 minutes were programmed for time spent using the tablet, and 109 minutes were programmed on day 2. The integration of individualized, tablet-based training into the BMT classroom environment provided a unique opportunity to reach at-risk groups with tailored content (eg, women and individuals who experienced sexual assault before enlistment), while protecting their privacy and providing consistent messaging to the larger group [14].

A separate SARC app containing individualized, tablet-based content for sexual assault survivors, which is too sensitive to deliver in a classroom setting, was made available for voluntary, confidential viewing in the SARC office following group training. Trainees followed BMT confidentiality processes to ensure they were not outed as a survivor if they voluntarily chose to receive this additional training.

In parallel with the training development process, we worked with partners at the BMT and Headquarters Air Force to determine the best approach for implementation and feasibility testing. The resulting plan included a gradual three-phase implementation that occurred over 26 weeks. Overall, 25% of incoming trainees received the SCC program, while the remaining 75% received the current BMT training in phase 1 (10 weeks). Moreover, 50% of incoming trainees received SCC during phase 2 (10 weeks), and 100% of incoming trainees received SCC during phase 3 (6 weeks).

All study procedures were subject to several rounds of Department of Defense (DoD) programmatic, legal, and human subjects protection review.

### Requirements of the Training Environment

Individualized, tailored trainings were at the forefront of both the application and technology design considerations when implementing the SCC project. Early in the development of the training approach, we learned that the trainees would have no access to personal or government-provided technology other than what was provided in the classroom. The nature of the SCC project was to do better than a *one-size-fits-all* solution; each trainee would receive tailored training targeted at them. The only 2 choices were web-based applications or mobile-based apps accessible by either tablets or laptops; the Air Force directed the use of Apple iPads and MacBooks.

Regardless of the technology, the trainees were to be restricted in what they could do with it. For example, during class, they were not permitted to access emails or surf the web. The device must only provide access to the apps pertaining to the classroom materials. This is analogous to *kiosk* or *single-app mode* where access to any other app is prevented.

In addition, we were informed that a Wi-Fi network providing access to the internet would not be available for our use; therefore, with no access to the internet, anything web-based was ruled out, including the use of a Mobile Device Management (MDM) system. An MDM allows administrators to remotely control and secure mobile devices such as tablets used in a classroom [15]. The lack of a network connection meant that there was no real-time access to a back-end storage server, such as SQL Server. As this more typical method of data collection and storage was not available, a nonstandard approach was required. Three months before the main study started, the project team was informed that limited Wi-Fi access was available, which resulted in a few minor changes to the system, but it was too late for the major changes required to take advantage of a networked system. Originally, because a network was not available, Verizon mobile hotspots [16] were incorporated into the system design to facilitate data transfer. With limited Wi-Fi access permitted, instead of using the Verizon mobile hotspot, the system was changed to permit MacBooks to connect directly to the limited Wi-Fi network as needed for data transfer. Modifying the BMT app to use a Wi-Fi internet connection was not realistic, given the project schedule.

The iPads were intended to remain in the classroom for reuse by other trainees undergoing the same SCC training. With classes in both the morning and afternoon, the devices had to maintain battery charge through the course of the day, with a full charge taking place overnight to be ready for the next day’s classes.

The SCC training was spread out over 2 days, with day 1 of SCC training occurring during week 2 of basic training and day 2 of SCC training occurring during week 4 of basic training. There was no guarantee that a trainee would be in the same classroom for their second day of training, and attempting to provide trainees with the same device they had from day 1 training was too high a burden on the instructional staff in addition to their other duties. Thus, 12 GB of content comprising all intervention programs had to be present on every device, as each use of the device could result in a different intervention program assignment for a trainee. Furthermore, with no real-time ability to access a back-end storage server such as an SQL Server database and all saved data needing to be anonymous to protect trainee privacy, a new approach was required for data collection and storage to link data over time and ensure trainees received the same program from day 1 to day 2.

### Objective

The aim of this study was to evaluate the efficacy and effectiveness of the technical implementation of the SCC program in a restricted educational setting with limited internet connectivity and the resulting ramifications. This is addressed by (1) how the technical solution met the requirements and (2) how it was implemented. We then review the technological feasibility results, discuss the lessons learned, and provide suggestions for different implementation strategies. The findings will provide guidance for future similarly restricted projects to focus on possible problems and alternative solutions.

## Methods

### Hardware

With guidance from the Air Force project team members, 364 iPads were purchased; 120 iPads were distributed to each of 3 classrooms. The remaining 4 iPads were distributed to the SARC office. Although each classroom has a capacity for 120 trainees, the average class size was less than 110, leaving at least 10 spare iPads per classroom to serve as backups in case something went wrong. In addition, 3 MacBooks were purchased, with 1 MacBook distributed to each classroom. To facilitate the charging and management of the iPads, we purchased 9 Evo 40 Cart [17]—Sync and Charge carts. Each cart connects, stores, and secures up to 40 iPads while also acting as a giant USB hub capable of providing simultaneous charging and synchronization, and 3 carts were stored in each classroom.

In the absence of Wi-Fi and consequently the inability to use an MDM, a rudimentary desktop MDM application, the Apple Configurator [18] was used to facilitate tablet management using Apple hardware. By physically connecting a classroom’s MacBook to the Evo 40 Cart, technical staff were able to configure and update the cart’s 40 iPads simultaneously before moving on to the next cart. After all, 3 classrooms were completed, 1 MacBook’s 4 USB ports were used to physically connect to, configure, and update the 4 iPads kept in the SARC office for voluntary supplementary training. In this way, while still a manual process, we were able to efficiently manage the 360 classroom iPads, replicating what could be done with an MDM if Wi-Fi had been available.

For security, each cart was physically locked, with the key stored in a combination lockbox accessible only by the project staff. The instructors distributed the required number of iPads based on the classroom roster before the class started. Distribution included headphones with disposable ear covers and single-use sanitization wipes to be used at the end of each training session. The iPads were already in single-app mode, also known as kiosk mode, preventing the trainee from accessing any other part of the iPad. Placing an iPad in a single-app mode was a task performed when the apps were installed or updated with a newer version.

To protect the MacBooks from unauthorized access, 2-factor authentication (2FA) was enforced. 2FA requires 2 methods to verify one’s identity: (1) something one knows, such as a username, a password, or a pin, and (2) something one has, such as a Personal Identity Verification, a security token, or a biometric factor [19]. As the system was not connected to the internal Air Force network, a government-provided Common Access Card (CAC) could not be used. Instead, a YubiKey [20] hardware security key was used as a physical authentication token.

### Custom Software

With the equipment design and setup complete, many of the technical requirements were met. To resolve the remaining technology requirements, we wrote custom software. Overall SCC program development, including content development, review, video production, and technical development and testing, took over 3 years.

Ensuring that the trainees received the appropriate tailored training on both days was challenging but key to the SCC project. With no way to ensure that a trainee would use the same iPad on both days, and no identifying data being recorded into a data storage mechanism, the project solved the problem of delivering the appropriate training on day 2 by implementing a *continuation code*. On day 1, upon completion of the screener instrument, the screening algorithm generated a 12-digit code. It was an obfuscated set of numbers (eg, 458-857-018-173) that embedded the information about their assigned intervention program. Trainees were instructed to write this continuation code in their BMT Study Guide. At the start of day 2 of the SCC program, trainees entered their continuation code to resume training. If a trainee did not have their continuation code (ie, they lost it, forgot their Study Guide, or never wrote it down), the app, with assistance from the instructor as necessary, directed the trainee to a *short screener*, a brief screening instrument that routed them to a self-identified sex-specific program that was least likely to be harmful and most likely to be appropriate.

To further protect trainee privacy during the class and because the single-app mode precluded the normal iPad lockout functionality, the SCC mobile apps implemented a custom lockout at the application level. Trainees were instructed to create their own 4-digit personal identification number (PIN) that could be used to lock and unlock the app throughout training. PINs were temporary and not saved with trainee data. The trainees created a new PIN for the day 2 class.

The use of the SCC apps resulted in deidentified data saved locally to each iPad. The Apple Configurator only provided the ability to manage iPads and update SCC apps with new versions and content. It did not provide the ability to extract data generated using SCC apps. Instead, custom software, designated the Data Aggregator, was written to connect and extract the data from the iPads to the MacBook and then aggregate the data into a format suitable for analysis. The use of the Data Aggregator is described in the Procedures Requiring Unique Skills section.

### Procedures Requiring Unique Skills

We tracked any classroom and system issues manually by reporting weekly with on-premises staff and via instructor and observer logs. In addition, use data were captured via the app, tracking how the trainee was using the app, which screens, the order of the screens, and how much time they spent on each screen they accessed within the app.

#### Daily Tasks

Before the start of each class, instructors placed iPads, headphones, disposable headphone covers, sanitization wipes, and classroom materials in each seat. At the end of each class, trainees wiped iPads with the provided sanitization wipes and disposed headphone ear covers. At the end of each class or training day, instructors collected the iPads, returned them to the carts, and connected the cables for each iPad to recharge for the next class.

#### Weekly Tasks

**Figure 1:**
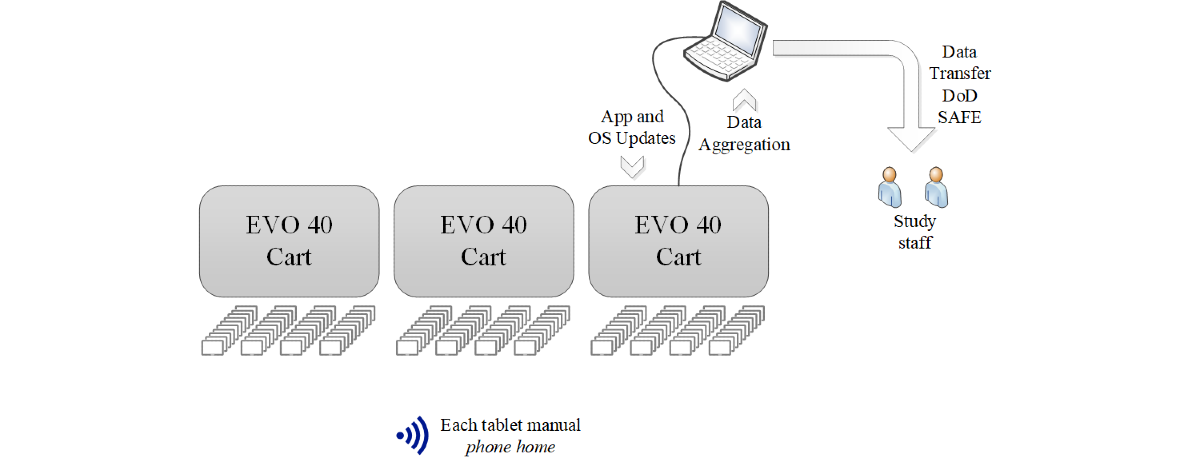
The classroom configuration of 3 Evo 40 carts, each cart containing 40 iPads. This figure illustrates some of the tasks performed by staff. This configuration was repeated in 2 other classrooms. DoD: Department of Defense; OS: operating system.

Every Friday, trained instructors connected the classroom’s MacBook to a cart and ran the Data Aggregator to pull the week’s data from iPads onto the MacBook (Figure 1). After repeating these steps for the remaining 2 carts in the classroom, the aggregated data were transferred to the project staff for study analysis via the DoD SAFE secure file transfer system.

The data collection steps were repeated in the other 2 classrooms. For the 4 iPads in the SARC office, because Wi-Fi was not available in the SARC office, 1 MacBook was brought to the SARC office, and all 4 iPads were connected directly to the MacBook. The same data synchronization steps were performed as in the classroom, and the iPads were then plugged back into their individual chargers, plugged into a wall power outlet. On returning to the classroom, the SARC app data were transferred to the project staff via DoD SAFE.

Backups of the data were kept on the MacBooks until it was deemed that the DoD SAFE transfer was successful and the data were processed. At that time, we deleted data from MacBooks.

#### Monthly and As-Needed Tasks

Apple requires software developers to use 1 of 2 Apple Developer programs. As these apps were internal Air Force apps, used only by Air Force personnel and not for distribution via the Apple App Store, we used the Apple Enterprise Developer Program [21] to generate distribution certificates. As part of fraud prevention, Apple requires each app to connect with Apple servers (ie, to “phone home”; Figure 1) to validate the authenticity of each app’s signing certificate. Under normal circumstances, mobile devices are regularly connected to the internet. With data plans, phones are permanently connected unless the user actively puts the phone in Airplane mode or turns off Wi-Fi and cellular data. On a tablet without a data plan, access to the internet is via Wi-Fi; this is how tablets are typically used. The required “phone home” for app certificate validation occurs behind the scenes, without the need for user intervention or notification. However, in this classroom setting, the lack of Wi-Fi access effectively prevented the phone home from automatically happening on the study tablets.

#### Operating System Updates

As per the instructions in the Authorization to Operate (described below), iPads and MacBooks required periodic operating system (OS) updates. All OS updates were first tested on the development test equipment to ensure field operability. Technical staff then traveled to the BMT to perform technical updates. The MacBook OS updates were performed first, followed by iPad OS updates (Figure 1).

With iPad OS updates, a phone home was also performed. Instead of each iPad downloading the OS update individually, an Apple Configurator was used to push the new OS to the iPads simultaneously. The classroom MacBook was first connected to an Evo 40 Cart. Using the Apple Configurator, the iPads were removed from the single-app mode and refreshed with the latest OS. The benefit of using the Apple Configurator for pushing the OS to the iPads was that the OS was downloaded only once per MacBook instead of 364 times (once per iPad). Once the iPads were updated, the OS setup was completed on each iPad. If required, a new version of the SCC app was pushed, and finally, a phone home was performed before putting the iPads back into single-app mode.

### Military Cybersecurity

Per US Air Force cybersecurity requirements, an Authorization to Operate (ATO) was required. An ATO is permission to use an information technology system. Working with Air Force cybersecurity personnel, appropriate security controls were identified that required documentation and possible software adjustments. Updates were entered into the Enterprise Mission Assurance Support Service (eMASS), a web-based application providing comprehensive cybersecurity management. Various cybersecurity-directed changes also needed to be applied to both iPads and MacBooks, such as turning off Bluetooth and enforcing 2FA.

### Conducting the Study

Although each technical task was meticulously documented and instructional staff received training in these tasks, the complexity and volume of the technical tasks, combined with the fact that the project technical team was off-site in another state, called for additional specialist safeguards. As such, BMT IT staff were also trained on technical tasks to provide on-premise support. The off-site technical project staff were on call during classroom sessions for troubleshooting and worked with on-premise IT staff to support the various technical tasks related to this project.

The feasibility study ran from September 2019 to March 2020, which was shortened by 4 weeks due to the onset of the COVID-19 pandemic. Approximately 800 new trainees start BMT every week, enabling a quick rollout of SCC to trainees. We received a non–human subjects research determination from the research team’s institutional review board, as the DoD deemed that the feasibility study was not research.

## Results

### Technology System

The technology system was tested with 190 classes, with 9196 trainees providing data on day 1 and 7742 providing data on day 2, resulting in at least 16,938 iPad uses. Owing to the COVID-19 pandemic, the study ended early; as a result, some day 1 trainees had never received day 2 training. None of the iPads broke or required Apple Care replacement; however, many headphones broke during repeated use (Table 1). Of the 7742 day 2 trainees, 758 did not have a working continuation code. More than 1000 unique data points were captured per trainee for scientific analysis with results forthcoming in future publications.

iPad issues during training, such as not turning on, freezing, needing to be restarted, or trainees being in the incorrect place in the training schedule, were noted by instructors for less than 14% of all class sessions. If a challenge was discovered during room setup, instructors borrowed unused tablets from the open classroom to ensure that all trainees would be able to participate in tablet content. Challenges that occurred during group instruction were managed by the co-instructor, while the primary instructor was able to continue instruction for the rest of the classroom; this was rendered more difficult if multiple trainees were having issues simultaneously. There were no difficulties with tablet accountability, distribution, recovery, storage, or data transfer.

Of the 190 classes conducted throughout the trial, there was only one issue that disrupted instruction. During the class, 80 tablets appeared to be “bricked,” completely unable to function. Confusing the issue was that of all the iPads, only 80 were nonfunctional. This disruption was compounded by a base-wide Wi-Fi outage that lasted several days, preventing the local BMT support team from troubleshooting potential solutions under the guidance of a remote technical team. On the day this problem was discovered, one class was unable to receive SCC training and subsequent classes. As the study was not yet running at 100%, we were able to borrow iPads from other classrooms until this issue was resolved. After a week of troubleshooting, we determined the malfunction was the result of Apple requiring a more frequent “phone home” than the 90-day frequency Apple recommended us to use. Detailed analysis showed that beginning approximately 6 weeks after the phone home, the iPads started to become unresponsive. However, not all 360 iPads became unresponsive on the same day. Rather, each day, a few iPads would randomly become unresponsive, until after a few weeks they were all unresponsive. Our tests could not establish a predictable pattern, and therefore, we performed the phone home connection every 28 days, thus guaranteeing that the iPads did not become unresponsive because of the inability to verify the certificate. We chose every 28 days to give us a 2-week buffer to the 6-week mark when we first noticed an unresponsive iPad in troubleshooting. We felt that this 2-week buffer was sufficient time to resolve any unforeseen technical issues, such as a Wi-Fi outage. The one class that did not receive the SCC training instead received the prior Sexual Assault Prevention and Response training that was already in place at BMT.

**Table 1:** The Sexual Communication and Consent technical issues tracked by the app and the instructor and observer logs.

Measure	Outcome	
	N	n (%)
Proportion of classes unable to continue because of tablet issue or systemic IT issue	190	1 (0.52)
Broken headphones	360	111 (30.83)
Did not have continuation code in day 2	7742	758 (09.79)

### Technical Activities

No analysis was performed to determine if the staff felt the system was too burdensome to maintain; rather, based on manual reports, we calculated the amount of time spent performing these technical activities. The instructors and BMT support staff engaged in the technical activities described in Table 2.

**Table 2:** The technical activities staff performed and the burden of each activity.

Activity			Commitment
**Training and ongoing technical assistance**			
	New instructor technical training	2 hours	
	Technical support with research team	1 hour per week; usually supporting data transmissions	
**Daily instructional time**			
	Setting up the classroom (including iPad distribution)	Up to 1 hour per class	
	Tearing down the classroom (including returning iPads to carts)	Up to 30 minutes per class	
**Classroom and tablet maintenance**			
	Weekly data transmissions to research team	Requires 1 person<30 minutes per classroom (3 classrooms total plus SARC^a^ office); dependent upon Wi-Fi	
	Monthly Apple license update (“phone home”)	6 hours; requires 2 people up to 2 hours each, per classroom (3 classrooms total plus SARC office); dependent upon Wi-Fi	
	Operating system updates (include app update and “phone home”)	Requires 2 people 6 hours per classroom (3 classrooms plus SARC office total); dependent upon Wi-Fi	

^a^SARC: Sexual Assault Response Coordinator.

## Discussion

### Principal Findings

This study showed that a modern, tablet-based, Wi-Fi–restricted classroom environment does work. We successfully implemented a tailored prevention program and collected data from over 7700 trainees over 2 training periods. With almost 17,000 uses, none of the 360 iPads broke or required a replacement from Apple via their Apple Care insurance. The SCC program was mentioned in the Fiscal Year 2020 Annual Report on Sexual Assault in the Military: Department of the Air Force as an innovative evidence-informed sexual assault prevention training as a new initiative to evolve the Department of the Air Force’s integrated prevention and response efforts [22].

Despite the promise of this innovative approach, we encountered several challenges that forced us to ask whether it is *worth it* to create such a custom environment. There is no disputing the importance of technological integration with scientific research, and with knowledge, we have gained better insights. In retrospect, the technology team should have pushed it harder for Wi-Fi access. As the SCC was custom, no other trainings could use the investment in the hardware the SCC required. In addition, the large cost investment in dollars and labor hours spent creating this custom solution are not feasible in most other environments. Understanding that importance is subjective, we present the following lessons learned in order of importance to SCC study.

### Lack of Wi-Fi

The number one issue that impacted many different parts of the system was the permanent access to Wi-Fi. Even with the rapid pace of innovation using technology, technology itself continues to be a barrier, with an internet connection being the largest [23].

Tight control over what and who accesses a network is integral to security. One approach to mitigate potential security concerns associated with allowing training iPads to access Wi-Fi would be to white list them, a mechanism that grants the IP address of each iPad explicit access to the Wi-Fi network. With permanent Wi-Fi access to the internet, the *phone home* challenge and related ramifications were eliminated. The hours spent troubleshooting why the iPads bricked in the first place before the 90-day marker and all the hours spent doing the *phone home* process every 28 days to 364 iPads would have been avoided. The study benefited from having a large total sample and missing one class, which did not affect the analysis, but if the N was much smaller, losing even one class could influence the data analysis. The *phone home* process was time consuming, averaging 6 hours to complete, and not without its own issues. Although the re-engineered process worked, manually connecting to Wi-Fi did not always work for the first time and sometimes required multiple attempts to forget and reconnect to the Wi-Fi network. White listing the iPads would have also prevented the need for multiple Wi-Fi connection attempts.

Access to Wi-Fi would have also permitted the mobile apps to save the data directly to a back-end cloud database storage server [24] for immediate access by project team members, a more standard architectural design for mobile app development. Instead, custom software had to be written, and trained staff had to be physically in the room to manually transfer the data from the iPads to a MacBook and then manually transfer the data to project team members via a secure file transfer mechanism.

Access to Wi-Fi would have also permitted the use of an MDM system such as IBM’s MaaS360 solution [25]. Instead of having trained staff physically in the room using MacBook client software manually connected to the iPads to manage them, an MDM solution would have permitted remote management of the iPads for OS updates and SCC app and content updates. MacBooks would have been used for emergency backup purposes only, as everything would be done remotely. Project staff, if remote, can multitask and perform other tasks while an update is being pushed. Without Wi-Fi, staff members were required to be in the classroom to monitor when the update was completed, sometimes waiting over an hour while the update was being performed.

Moving to a web-based solution for the training still requires Wi-Fi but eliminates the need for MDM to manage this specific class. This structure of this study was for the trainees to only use the iPad during the SCC class in a classroom setting. The iPads were stored in the classroom and were only accessed within the classroom. The SCC training was only one class of many as the trainees went through basic military training. If the overall education system for basic training is invested in Wi-Fi infrastructure and more permanent use of technology by trainees, SCC could become part of that ecosystem, eliminating the need for a stand-alone system that cannot be used by any other training. Although an MDM would still be needed for the overall management of the device, it would be integrated into the overall educational ecosystem and no longer be SCC-specific.

### Procedures Requiring Unique Skills

As the study progressed, the instructors and BMT IT staff became more comfortable performing technical activities (Table 2) required of them.

Troubleshooting via text messaging and videoconferencing between BMT IT and instructor staff with remote research and technical staff was very efficient. Although the cellular connection was not always stable, the ability to *see* what was happening from a remote location streamlined troubleshooting greatly. Remote staff can direct the premises staff much more easily and make real-time suggestions instead of waiting for emails to go back and forth. There was a need for an IT person to be *available* while classes were in session should something go awry. During the study, the remote project staff maintained an on-call approach, which was very useful. During the study, instructors completed many of these IT tasks and became proficient in the procedures and self-sufficient within a few weeks in the field. Instructors who were already familiar with the Apple ecosystem became especially proficient. On the basis of SMS text message and FaceTime history, there appeared to be a decrease in both forms of communication as the study progressed.

Each class required 1 hour and 30 minutes of setup and teardown time. Having dedicated classrooms permanently provisioned to avoid setup and takedown procedures could significantly reduce the daily manpower requirements. Instructors and IT staff could divide classroom and tablet maintenance tasks to disperse the associated manpower requirement.

Every 28 days, staff members were required to perform the monthly Apple license update (“phone home”) task. The regularity with which this task needs to be performed is not found in public Apple documentation. If the application does not connect, it becomes unavailable for use on the device. As the iPads were in single-app mode, the result of this unavailability manifests as an unresponsive tablet with a blank screen and no error messaging.

To avoid this, and because the tablets could not automatically phone home, instructors were trained to perform this license validation task manually on each iPad. Per Apple direction, we trained instructors to perform this certificate validation task every 90 days.

Critical to the study’s success were other skill sets, including staff with expertise in the Apple ecosystem, including MacOS, iPadOS, MacBook, and tablet maintenance; software development skills for app updates and OS updates; and Air Force cybersecurity requirements to maintain the ATO. When implementing this program, or others like it, in the future, staff will need to commit sufficient time to ensure that the program runs smoothly on the tablets (eg, by conducting quality checks after each device or app update). Software development troubleshooting skills are highly recommended because of the nature of mobile software updates [26].

Owing to their intimate knowledge of the study’s technical ecosystem, having a software developer as part of the support team was key. In addition to maintaining the annual Apple Developer Enterprise Program License yearly, they were able to troubleshoot app issues that may be introduced through iOS updates (eg, someone who knows what to do if the keyboard comes upside down on the tablets after an automatic system upgrade) and rebuild and redeploy the app to all iPads when needed.

The instructors, through training and use of the system, became technical experts on the system. They needed ample lead time in the classroom and support to work with the technology and became well versed in addressing problems that arose with iPads and the app during classes. Building trust among all staff members is important when conducting research using unfamiliar technology. Unfamiliarity can breed uncertainty, and SCC benefited from open, early, and frequent communication among all team members. Weekly technical assistance calls were set up to address any questions or concerns that the instructors had. Instructors should not have to become technology experts in their classroom; rather, they should be able to use the technology and focus on engaging with students, especially in sensitive topic areas, such as sexual assault, which can trigger an unexpected emotional response.

### Broken Headphones

Headphones breaking over time was reported as an issue. Headphone reuse and sanitation worked for this class, but due to the constant bending of headphones to place over the ears almost 17,000 times, approximately one-third of the headphones needed to be replaced (Table 1). The dilemma was whether to purchase many cheap headphones frequently or more expensive headphones that were more durable and lasted longer. No determination was made in this study that comparing cheaper quality versus higher quality headphones other than projects using headphones should plan to replace headphones over time.

### Hardware Choices

What about using Google Android instead of Apple iOS or iPadOS in the future? Using non-Apple equipment was not an option; the requirement was to use technology from the Apple ecosystem, following the trend of other Air Force projects using Apple technology. However, switching to an Android ecosystem would only, perhaps, save on the phone home. Our experience on other projects using Android indicates that there are no phone home requirements for Android applications. Although avoiding the phone home issue would have saved labor and time, all the other issues would have remained as the issue was around Wi-Fi access, not what OS the mobile device was running.

If trainees had iPads assigned to them, perhaps at the start of BMT, the need for a continuation code could have been avoided. The continuation code, a set of obfuscated numbers that embedded information about an assigned intervention program, was more an annoyance than a problem. All data were deidentified, and with no back-end system to query, it was impossible to know what intervention program the trainee was assigned to. This was crucial to the study design for trainees to receive correct tailored training.

The Evo sync-n-charge cart is indispensable for efficiently managing manual technical tasks and charging. In addition to charging the iPads, when a MacBook was connected to the cart, all 40 iPads were available for bulk updating via Apple Configurator software. The Apple Configurator worked well as a manual MDM, with the only downside being a lack of detailed progress. For example, when pushing 12 GB of SCC content to 40 iPads, there was no indicator of how much content was pushed to the number of iPads. Only through trial and error did we determine that the software was not hung up but rather, with *started* and *completed* status updates as our guides, we learned it took 3 to 4 hours to update 40 iPads with all the SCC content.

### Lack of Data Storage Lookup Mechanism

As the system had no back-end data storage mechanism, there was no automated solution to look up a trainee’s assigned intervention program. The continuation code solution was an annoyance because trainees had to write down a long, 12-digit number and then key it into the app on day 2. Of the 7742 day 2 trainees, 758 did not have a working continuation code (Table 1). If the trainee did not have the code from day 1, they would complete a short questionnaire asking for self-identified sex only. On day 1, the full screener placed the trainee in 1 of 5 different intervention programs. This shortened questionnaire would place the trainee into 1 of 2 intervention programs as time did not permit the trainee to go through the entire screener again. The potential risk is that the trainee would not receive the same tailored program.

If trainees had iPads assigned to them, perhaps at the start of BMT, the need to have a continuation code could have been avoided. The data would be saved locally on their assigned iPad, including the correct intervention program assignment. In mobile apps, extra software development for the error handling of a lost continuation code could have been reduced. More importantly, this would also reduce the risk of trainees receiving a different intervention program.

### Military Cybersecurity

The use of mobile technology was new to the BMT classroom at the time, and for security purposes, the military was rightly cautious in its adoption of new technologies. Conducting research with the military provides its own set of unique challenges with many required signoffs. Research using IT should plan on a Certified Information Security Manager (CISM) to assist with ATO. The CISM interfaces with the cybersecurity team at the military and submits all documentation into eMASS. An experienced IT technical team works with CISM to determine relevant security controls and Security Technical Implementation Guides to implement. The IT team needs to plan for extra time to update the design and implement changes in the system as per the Security Technical Implementation Guides.

Owing to the nature of the unique SCC system being a closed system with no cloud access, many of the security controls were not applicable, which prompted some discussion on how to accurately record the correct response within the eMASS system. This study broke new ground, and electronic systems were not set up to track cybersecurity details efficiently. Additional details are required and documented in the security control documentation because of the custom solution of the SCC system.

The SCC operated under an interim authority to test, then a 1-year ATO, and then a 3-year ATO was issued. Project planning should account for the appropriate time in the schedule to perform ATO-related tasks: where the system will be hosted, updating the system with the required changes, and documenting the security controls in eMASS.

Finally, projects should plan for staff members to pass a security clearance and obtain CAC and government-issued computers to access eMASS. A CAC is a smart card and the standard identification used by the DoD for physical access to buildings, controlled spaces, and access to computer networks and systems. Owing to circumstances beyond our control, it took 13 months for the project staff to receive their CACs after the initial security clearance submission.

### Next Steps

The SCC project team has since developed a web-based version of the SCC program to resolve many of the challenges faced in this initial study. We are currently testing and evaluating this version of the program in a military setting that is Wi-Fi enabled. In addition, we plan to evaluate the integration of the SCC program into standardized web-based e-learning systems in other military locations.

### Conclusions

The use of mobile technology in a classroom setting allows for new ways of teaching, especially delivering training tailored to a specific student. Although the use of technology in new ways is exciting, it presents new challenges and requires new skills. The SCC project shows that mobile technology in a unique, Wi-Fi–restricted classroom setting does work, but that improvements are needed before it will be sustainable or scalable. Testing any system in its intended environment is critical for increasing its acceptability, feasibility, and usability [27]. Advances in technology allow for new and creative ways of conducting scientific research, and we encourage researchers and technologists to explore new uses of technology. Considering the constraints and lessons learned in this study will impact overall technical implementation.

## Abbreviations

ATO: Authorization to Operate
BMT: Basic Military Training
CAC: Common Access Card
CISM: Certified Information Security Manager
DoD: Department of Defense
eMASS: Enterprise Mission Assurance Support Service
MDM: Mobile Device Management
OS: operating system
PIN: personal identification number
SARC: Sexual Assault Response Coordinator
SCC: Sexual Communication and Consent
